# Pilot of a Community Health Worker Video Intervention for Immigrant Day Laborers at Occupational Health Risk

**DOI:** 10.3389/fpubh.2021.662439

**Published:** 2021-07-22

**Authors:** Marielena Lara, Claudia Díaz Fuentes, Jorge Calderón, Sandy Geschwind, Meshawn Tarver, Bing Han

**Affiliations:** ^1^RAND Corporation, Santa Monica, CA, United States; ^2^Department of Economics, University of New Mexico, Albuquerque, NM, United States; ^3^Common Ground Health Clinic, New Orleans, LA, United States; ^4^Department of Research and Evaluation, Kaiser Permanente Southern California, Pasadena, CA, United States

**Keywords:** immigrant, day laborer, Latino (Hispanic), video intervention, promotor, *promotora*, occupational health risks, preventive behaviors

## Abstract

**Significance:** Immigrant day laborers suffer from disproportionate occupational health risks from hazardous reconstruction jobs after natural disasters.

**Methods:** We conducted a randomized controlled trial of a short-video educational intervention to improve safety knowledge and intent to engage in safety preventive behaviors among 98 Hispanic day laborers (49 randomized to video and 49 control). The short video featured a male *promotor* and a female *promotora* who narrated 3 stories of day laborers who were injured while doing construction work in post-Katrina New Orleans. The main outcome measures were changes in scores for day laborer-reported safety knowledge and safety behaviors derived from interviewer-delivered baseline and post-intervention surveys.

**Results:** Video participants reported improvement in overall average safety knowledge score (mean score of 11.3 out of a max score of 12 or 94% when standardized to 0–100% scale), as compared to the control group (mean score of 8.6 or 72%) who were not offered the video (*p* < 0.00001). The intervention was highly successful in workers stating that they learned and were willing to change their safety preventive behaviors to reduce their occupational risk. The average safety behavior score was higher among those watching the video (17.2 out of a max of 22 or 78.1% when standardized on a scale 0–100%) as compared to control (14.5 or 65.9%) (*p* = 0.0024).

**Conclusion:** A short video intervention can improve knowledge and intent to engage in preventive behaviors among Hispanic workers for which there is a dearth of construction safety preventive research.

## Introduction

The United States (US) National Institute of Occupational Safety and Health (NIOSH) has designated immigrant Hispanic construction workers as a high-risk and high-need population. After agriculture, the construction industry has the second largest proportion of Hispanic workers with a higher rate of fatal (1, 2) and non-fatal occupational injuries (3–5), as compared to their white and black counterparts (6). These disparities are heightened by the fact that construction-related injury rates are substantially underreported among Hispanics (3, 7, 8).

US immigrant Hispanic day laborers are often targeted by contractors who do not comply with the US Occupational Safety and Health Administration (OSHA)'s occupational injury prevention policies, picking them up from street corners for dangerous jobs with no written guarantee of pay (9). Although OSHA policies protect all workers independent of immigration or legal status, fear of deportation can hinder day laborers from reporting unsafe working conditions and accessing health care for occupational-related injuries (10). Partnerships with providers engaged with these communities allow implementing occupational programs to help prevent, and if necessary treat, day laborer's injuries in a context of respect for patient's privacy and trust to not release immigration status information.

Immigrant day laborers conduct a disproportionate amount of reconstruction involving demolition, cleanup of debris, and other potentially hazardous jobs after natural disasters. For example, after Hurricane Katrina devastated much of the New Orleans, Louisiana, a large numbers of predominantly young, uneducated and undocumented Latino migrants arrived in the city to help (11–13). The increasing frequency of natural disasters and related demand for day laborers underscores the need for evidence-based strategies to help prevent and manage the health effects of the occupational environmental exposures experienced by this vulnerable group. There is, however, not enough construction safety and health research among Spanish-speaking workers with low literacy, in general, and interventions to improve occupational prevention knowledge and behaviors, specifically (1, 4, 5, 14–17).

Prior research suggests viable interventions for vulnerable populations (14–20). For instance, lay or community health workers (male *promotores* or female *promotoras*) knowledgeable about the community have been effective at translating evidence-based preventive health care and interventions for underserved populations (4, 18, 19). Safety coaching by construction site foremen that include daily verbal exchanges with workers has been also shown to have a significantly positive effect on safety levels (20).

Likewise, video interventions are also effective in improving prevention knowledge and behavioral intent in other conditions and populations (21, 22). One benefit of short videos as a preventive educational strategy is that they can be easily and inexpensively shown in clinics' waiting rooms at community health centers attended by the target population (23). This is in comparison to in-person trainings that have been demonstrated to improve worker' knowledge, attitudes, preventive behaviors, and self-efficacy, but last over 4 h (4, 24, 25).

Other work highlights the potential benefit of a combined peer-video approach, reporting that *promotora-*delivered video and written materials are effective in changing pesticide-related behaviors, with *promotora* attributes contributing to intervention success (26, 27). Formative work conducted by the investigators (28) and others (29) supported the potential value of a combined peer-video health intervention to reduce occupational health risks faced by day laborers. For this pilot study we chose a *promotor(a)*-led short-video format, instead of an in-person *promotor(a)* intervention, because formative work showed that a video format was more feasible, and could allow for the intervention to be replicated in other settings and to be disseminated to a larger number of lower literacy populations.

The goal of this study was to develop and pilot a *promotor(a)-*based short-video intervention to reduce occupational health risks faced by Hispanic workers in the US. Specifically, the research aimed to find out whether a brief educational video featuring a male *promotor* and female *promotora* can increase safety knowledge and intended use of safety preventive behaviors regarding occupational risks among Hispanic day laborers.

## Methods

We used community-based participatory research (CBPR) methods to develop and pilot a brief video *promotor(a)* intervention for the Hispanic worker population to improve safety knowledge and use of safety preventive behaviors regarding construction occupational risks. We used a randomized and controlled approach to provide preliminary data on the efficacy of the video intervention. Interviewer-delivered baseline and immediately post-intervention surveys were conducted to estimate short-term changes in safety knowledge and intended safety behaviors.

The content of the intervention was informed by a formative assessment process that documented the occupational exposures and experiences of day laborers, as well as their perceptions on existing educational materials available in Spanish through OSHA's website (28). The video script was developed in Spanish through an iterative and collaborative process involving the staff at Common Ground Health Clinic (CGHC), an industrial hygienist, a Hispanic videographer, the day laborers who featured in it, and the native Spanish-speaker investigators with experience in video production. CGHC identified and recruited the featured day laborer *promotor(a)*. The recruitment criteria were being a day laborer who was currently working in that capacity, had direct knowledge of the community, was fluent in Spanish, and very interested in working with fellow day laborers and the research team. Once recruited, the investigators trained the day laborers about the project following a standardized protocol.

The resulting intervention was a short educational video that highlighted best practices regarding occupational hazards and preventive behaviors identified as most relevant during the formative assessment stage. The video included three vignettes about roofing, ladder falls, as well as demolition and clean up, correspondingly. In each vignette, the *promotor(a)* demonstrated the appropriate equipment, a potentially risky situation, and modeled safety behavior. The video closed with a summary of three actions day laborers can take to avoid risky situations, and resources to report unsafe working conditions. The editing process involved final selection of the video clips and messages to balance camera time of both the male *promotor* and the female *promotora*, visual and cultural appeal of the images, and content to cover the key safety occupational knowledge and preventive actions. Final cuts kept the video under 12 min.

### Participants

The target population was Spanish-speaking day laborers in the US who work in the construction industry, for whom there is a dearth of appropriately language- and culturally appropriate occupational information. The community partner (Common Ground Health Clinic or CGHC) led the recruitment process from their main clinic site, and their mobile preventive health clinic that meets migrant workers at major job search areas. Approached day laborers received a brief description of the study and were eligible if (i) 18 or older, (ii) preferred Spanish, (iii) had worked as a day laborer in the past 12 months, and (iv) planned to work as a day laborer in the next 3 months.

### Study Procedures

Eligible individuals who were interested in participating completed written informed consent in Spanish before proceeding to answer the baseline survey. Once the baseline survey was completed, the data collector opened the sealed envelope that determined if the subject would be assigned to the video or control group. Participants randomized for the treatment group (video) watched the video in a separate location with no distractions. Once it was completed, participants responded to the follow up survey. Participants randomized to the control group took a 15–30 refreshment minute break before answering the follow up survey and then given the option to observe the video. The Human Subjects Protection Committee at the RAND Corporation approved this study. A Certificate of Confidentiality to protect information about the subject's identity and immigration status was granted by the US Department of Health and Human Services.

### Randomization

We implemented a blocked randomization strategy where blocks were recruitment sites (work locations, clinic). A random sequence of study condition assignments was drawn before the beginning of the recruitment in each block. The sequences ensured that the sample sizes in each condition would be approximately balanced. Recruiters were blind to the randomization sequences.

### Sample Size

A total of 100 participants were approached and recruited for the study's two arms. Two participants left the study before completing the baseline survey. Our final sample, therefore, included 98 individuals, 49 randomized to the treatment group (video) and 49 randomized to the control group. All participants remained in the study for the post-intervention survey. As a pilot study the planned sample size was not meant to detect a small treatment effect but to provide a crude estimate of the possible effect size. The planned sample size (*n* = 50 per arm) can yield a 95% confidence interval with a half-length of 0.28 times standard deviation, or equivalently, have a power of 80% to detect a standard effect size of 0.57 times standard deviation with a two-sided *p*-value < 0.05.

### Survey Measures

The baseline survey included items for demographic characteristics, type of job in past 3 months (e.g., demolition, roofing, etc.), literacy level (30), type and frequency of health exposures, access to safety training, and knowledge about occupational safety. The baseline and post-intervention surveys included items to evaluate changes in knowledge (Table 1) and intent to engage in preventive safety behaviors (Table 2). The knowledge items were coded as 1 if the responded answered correctly (options were true or false) and 0 otherwise. The Summary Safety Knowledge Score constituted an aggregate score ranging between 0 and 12. Safety behaviors were coded as binary variables set to equal 1 if the participant intended to invariably engage in a safety behavior and 0 otherwise. The Summary Behavior Score that summed all the knowledge items ranged between 0 and 22. Three behavior sub-scores were also created and calculated: (i) behaviors related to self-advocacy (between 0 and 5), (ii) other protective actions (between 0 and 9), and (iii) use of protective equipment (between 0 and 8).

**Table 1:** Preventive occupational knowledge associated with video intervention.

**Baseline and follow up survey items**
**Answer options: true/false**
**Summary safety knowledge score (items** **=** **12)**
Your employer needs to inform you about your occupational risks.
Before starting a job, workers must identify occupational risks.
Workers should try to obtain their own protective safety equipment.
Workers may refuse to take dangerous jobs without any consequence or punishment.
OSHA only protects workers who are legally in the United States.
Mold can cause itchiness, allergies and respiratory problems like asthma.
A rag protects like a mask or respirator.
Masks to protect from gases and vapors are different from masks that protect from dust.
When cleaning mold, it is recommended to use an N95 mask.
It is necessary to use a filter mask when spray painting.
A disposable full body suit can be used if exposed to dust or splashes.
Hard hat is always needed in construction.

**Table 2:** Preventive occupational safety behaviors associated with video intervention.

**Answer options: Likert scales dichotomized if participant engaged in safety behavior (baseline survey) or intended to do so (follow up survey)**
**Summary safety behavior score (items** **=** **22)**
**Self-advocacy behaviors sub-score (items** **=** **5)**
Never takes a job that is risky or dangerous
Ask the employer about the possible dangers
Ask the employer for the proper safety equipment
Never avoids asking for equipment for fear of being fired
Calls OSHA to report danger
**Protective actions sub-score (items** **=** **9)**
Do a head to toe check to make sure that you are protected by correctly using the correct equipment
Buy your own personal safety equipment if your employer does not provide it
If buy own protective equipment won't have enough money for other needs
If using a ladder, make sure it is well-placed and stable
Check the area before working to identify any danger
Try to make sure there is air flow or the appropriate ventilation
Check to make sure there is no danger of falling
When working on remodeling and other places where there could be mold, clean the area with diluted bleach
Gather all materials damaged by mold and dispose in bags
**Protective equipment use sub-score (items** **=** **8)**
Disposable mask
Respirator with filter
Harness
Strong gloves
Goggles/glasses
Ear protection
Hard hat
Steel toe boots

### Survey Development

The surveys' language and cultural appropriateness were evaluated by a committee of bilingual and bicultural Spanish native speakers. The survey(s) were piloted in *n* = 8 individuals to assess understandability, literacy appropriateness, and time required. The design and modifications to the survey items or instructions made as a result of pilot testing were conducted in Spanish and then back-translated into English following a language and cultural equivalence consensus approach.

### Statistical Analysis

The success of the randomization was assessed comparing treatment and control groups at baseline for demographic characteristics, type of jobs, knowledge and safety behaviors using a *t*-test of difference in proportions of independent groups. The evaluation of treatment effectiveness used the same statistical technique but comparing pre and post-treatment differences in mean scores between the groups.

## Results

### Sociodemographic and Occupational Characteristics

Table 3 shows key demographic and occupational characteristics of participants randomized in treatment and control groups. Overall, the two groups had comparable socio demographic attributes with almost all participants being male, preferring Spanish with an average age of 40. About 60% of participants had less than a high school education. In terms of literacy, the mean score was 14.5 with over 60% of participants meeting the 15-point cutoff for adequate literacy (30).

**Table 3:** Participant characteristics by study arm.

	***n***	**Treatment**	**Control**
**Sociodemographic characteristics**			
Sex (Male, %)	97	93.9%	97.9%
Age (Mean)	89	40.3	40.5
Married or living together (%)	97	53.1%	33.3%
Race non-white (%)	95	27.7%	29.2%
Prefers Spanish (%)	96	91.7%	89.6%
Country of origin (%)^++^			
Honduras	97	46.9%	45.8%
Mexico^*^	97	30.6%	12.5%
Guatemala	97	6.1%	16.7%
Other	97	16.3%	25.0%
Level of education (%)			
No schooling	97	6.1%	6.3%
1–5 years	97	20.4%	16.7%
6 years	97	14.3%	14.6%
7–11 years	97	24.5%	16.7%
High school or more	97	34.7%	47.5%
**Baseline occupational characteristics**			
In the last 3 months, have you worked in jobs…			
With gases or smells that made you cough?	98	28.6%	34.7%
That left you with skin rash or itchiness?	98	42.9%	42.9%
That gave you back pain?	98	46.9%	59.2%
In the last 3 months, have you…			
Worked on surfaces that are over 6 ft (2 m) from the ground?	98	85.7%	77.6%
Worked in places with loud noises?	93	65.2%	63.8%
Have you ever received training by an employer or anyone about…?			
How to use a harness?	98	49.0%	49.0%
How to use a mask or a respirator?	98	55.1%	51.0%
Hazardous substances or chemicals?	97	39.6%	24.5%
How to protect yourself from mold?	95	25.0%	36.2%

* *There was no statistically significant difference in occupational characteristics of individuals in the treatment and control groups (p < 0.05). In sociodemographics, the only statistically significant difference was being born in Mexico. Significance of difference in means for discrete variables was calculated using t-test for difference in proportions*.

++ *Other countries include El Salvador, Puerto Rico, Cuba, and United States*.

Regarding occupational characteristics, participants performed, on average, three different types of jobs at the construction site with painting (61%), roofing (34%), demolition (34%), and framing (29%), among the most prevalent. Among all participants, the 3 more common job-related reported symptoms were back pain, skin rash or itchiness, and ear, nose or throat irritation. The most commonly reported respiratory hazards listed were dust from cement, concrete, tile, etc. (76.5%), mold (39%) and lead (37.3%). In terms of risky occupational activities, participants reported working on surfaces 6 feet or more above the ground (81.6%), cutting wood or other materials (78.6%), and half reported working on their knees for long periods of time or in working without protection in places where something could fall on their head.

About half of participants reported having received training about how to use a mask or respirator, use a harness, safety googles, and portable ladders. Two thirds said they had not received training about hazardous substances or chemicals, what to do about inappropriate ventilation for a job, and to protect themselves from asbestos or mold. Among those reporting training, about one third said they received training from co-workers, family or friends, health professionals, or other community organizations, and not employers, although employers are required by law to do so.

### Improvements in Preventive Occupational Knowledge Associated With the Video Intervention

At baseline the treatment and control groups were comparable in terms of the Summary Knowledge Score (max = 12) as well as the item-specific knowledge items shown in Table 1. At baseline participants in both groups reported a higher than anticipated occupational knowledge with nearly 60% of participants answering 10 out 12 questions correctly. The exception was lack of knowledge about how OSHA protects workers independent of their legal status in the US, with only close to half of respondents reporting knowing this at baseline.

The video *promotor(a)* intervention was associated with statistically significant improvement in occupational preventive knowledge both overall and by specific content areas (Table 4). The treatment participants (video) reported improvement in overall average knowledge score (mean score of 11.2 out of 12), as compared to control group (mean score of 8.6) who were not offered the video. Other significant improvements regarding knowledge were day laborers' rights to: (i) refuse dangerous jobs, (ii) obtain protective equipment, and (iii) be protected by OSHA even if they are not legally in the US (*p* < 0.01), and (iv) effectiveness of different types of protective equipment [e.g., rags do not protect like a mask or respirator (*p* < 0.05)].

**Table 4:** Means and changes in preventive occupational behaviors and knowledge scores.

	**Baseline treatment *n* = 48**	**Baseline control*n* = 48**	***P*-value**	**Follow-up treatment*n* = 48**	**Follow-up control*n* = 48**	*p*-value^*^
**Summary safety knowledge score** ***(*****max** **=** **12*****)***	**9.898**	**9.3061**	**0.1539**	**11.245**	8.612^*^	**0.000**
**Summary safety behavior score (max** **=** **22)**	**10.265**	**10.551**	**0.716**	**17.184**	14.510^*^	**0.003**
Self-advocacy behaviors sub-score (max = 5)	1.673	1.776	0.657	3.612	2.939^*^	0.026
Protective actions sub-score (max = 9)	3.918	4.245	0.473	7.000	5.551^*^	0.004
Protective equipment use sub-score (max = 8)	4.673	4.531	0.756	6.571	6.020	0.173

* *Statistical significance between treatment and control groups at p-value < 0.05. The important subcategories of the results are bolded*.

### Improvements in Intended Preventive Occupational Behaviors Associated With the Video Intervention

The video intervention was highly successful in workers stating that they learned and were willing to change their behaviors to reduce their occupational risk. The average behavior score was statistically significantly higher among those watching the video (17.2 out of a max of 22) compared to control (14.5 points) (Table 4). Results from the subscales for the behavior-specific items shown in Table 4 showed improvement for the treatment group as compared to the control group (*p* < 0.05): (i) “Self-advocacy” behaviors (i.e., turning down a dangerous job, asking employers about possible dangers, calling OSHA to report danger), and (ii) “Other Protective Actions” (i.e., checking their own protective equipment head to toe, inspecting an area for possible risks before starting work, using a ladder correctly, making sure there is adequate ventilation, appropriate cleaning and disposal of mold infested materials).

## Discussion

We piloted a short-video educational intervention to improve construction-related risk knowledge and intent to engage in preventive safety behaviors among Spanish-speaking day laborers who experience occupational-related disparities. The video presentation featuring a male *promotor* and a female *promotora* was highly successful in improving reports of safety knowledge gained and workers stating that they would change safety behaviors to reduce their occupational risks. The *promotor(a)* played the role of day laborers as they narrated 3 stories of workers who got injured while doing day construction work. Once they saw the video participants reported improvement in overall average safety knowledge score as compared to the control group who were not offered the video. The video intervention was highly successful in workers stating that they learned and were willing to change their behaviors to reduce their occupational risk, with the average safety behavior score being statistically significantly higher among those watching the video as compared to control.

Our findings are consistent with previous work that supports the benefits of a participatory approach in designing educational videos for both occupational (29, 31, 32) and other preventive medicine interventions (21, 22). We believe that integrating *promotores(as)* in both the development of the script and as actors in the video was paramount in the efficacy of the video with the intended audience. Likewise, working with a Hispanic videographer promoted the use of culturally-congruent messaging in the execution of the video scenes. Although it took time to find a Hispanic videographer and *promotor(a)* who had the necessary technical skills, the success of the product depended on them being part of the research team.

We also replicated the beneficial role of peer-support approaches in difficult-to-reach populations (33, 34), and specifically, engagement with a *promotor(a)* in preventive medicine interventions to reduce occupational exposures (26, 35). In the specific case of occupational health, working with *promotores(as)* to reach workers in high-risk jobs eases access and acceptance, particularly when workers are not protected by standard work arrangements (such as unions and benefits), or move worksites regularly to maintain employability (35). Working with *promotores(as)* as cultural and community gatekeepers and mediators greatly enhanced recruitment for and implementation of our pilot study.

Our approach to recruiting and training *promotores(as)* through our community partners is well-established in the literature (27, 36). CGHC identified Spanish-speaking day laborers who had direct knowledge of the community, were engaged in construction work and were aware of OSHA safety regulations. Previous research documents the common use of both formally trained community health workers (35), as well as individuals from the target population with knowledge of the community and commitment to public health to help develop and deliver a culturally sensitive and relevant intervention (33, 37). The partnerships with a local community clinic trusted by the day laborers also was also key strength.

Although previous research on peer-developed video interventions among immigrants is rare (22, 31, 32). and generally focused on farmworkers, our results support other work showing that peer-developed video-based interventions are effective in improving safety behaviors and safety knowledge (31, 32, 36). Our video also incorporated elements of empowerment by emphasizing knowledge of the applicability of OSHA regulations to all day laborers independent of immigration status. The *promotor(a)* in the video role modeled how day laborers can stand up to their employers when protective equipment is not provided. Although empowerment-like approaches have been used in longer in-person training (more than 4 h) (24, 25), to the best of our knowledge they have not been implemented in short videos like our own.

Although the randomized and controlled design for this pilot strengthens our findings, there are several limitations that are important to consider in the interpretation of our results. The sample size was slightly lower than planned. Although in the end we had enough power to detect moderate effect sizes, the small sample size limited the number of outcomes we could evaluate. It was also not feasible to assess retention of gained knowledge beyond the immediate intervention period, nor associated changes in actual self-protective behaviors, instead of intended behaviors. Finally, although the results of this pilot study provide a good case study for an effective prevention intervention in a group of post-Katrina day laborers, the findings may not be generalizable to construction worker populations whose work is non-transient, though it provides relevant insights for day laborers in other industries. Acknowledging these limitations, we speculate, however, that implementation of the video intervention in a community setting could contribute to favorable end outcomes—increased actual safety preventive actions and associated reductions in work-related exposures, morbidity, and mortality—among other Hispanic day laborers.

## Conclusion

This study showed that a brief educational video featuring a male *(promotor)* and female *(promotora)* can increase safety knowledge and intended use of safety preventive behaviors regarding occupational risks among Hispanic day laborers. In doing so, it contributed new knowledge about the effectiveness of *promotor(a)* interventions, in general, and video interventions, specifically, to present culturally, linguistically, and educationally-appropriate methods of communication to inform difficult to reach and vulnerable populations. Lessons learned about the effectiveness of the pilot intervention in post-Katrina New Orleans can inform other interventions to reduce occupational risk in Hispanic and other vulnerable workers responding to natural disasters in other locations.

The results of this pilot study also increased our understanding of working conditions experienced by US Hispanic day laborers after a natural disaster, for which there is a dearth of construction safety preventive research. From a research perspective, the next step would be scaling up the intervention and conducting a larger-scale effectiveness trial to replicate the findings and follow up workers to assess, not only changes in knowledge and behavior intentions, but also the long-term impact on work safety behavior.

## Data Availability Statement

The raw data supporting the conclusions of this article will be made available by the authors, without undue reservation.

## Ethics Statement

The studies involving human participants were reviewed and approved by Human Subjects Protection Committee at the RAND Corporation (Assurance number FWA00003425, IRB number IRB00000051). The patients/participants provided their written informed consent to participate in this study.

## Author Contributions

ML was the PI of the project and oversaw the design, implementation, analysis and write up of results presented here. CD co-developed the survey, analyzed and drafter results. JC recruited, interviewed and assisted in data analysis. SG co-developed the intervention, script for the video, contributed to the review of literature and survey design. MT, then director of Common Ground Health Clinic, coordinated the entire field work for data collection and intervention implementation. She was instrumental in the design of the intervention protocol and survey. BH developed the sampling strategy and co-developed the analytical strategy for the results presented here. All authors contributed to the article and approved the submitted version.

## Conflict of Interest

The authors declare that the research was conducted in the absence of any commercial or financial relationships that could be construed as a potential conflict of interest.

## References

[1] Dong X Platner JW. Occupational fatalities of Hispanic construction workers from 1992 to 2000. Am J Indus Med. (2004) 45:45–54. 10.1002/ajim.10322 14691968

[2] Dong XS Fujimoto A Ringen K Men Y. Fatal falls among Hispanic construction workers. Accident Anal Prevent. (2009) 41:1047–52. 10.1016/j.aap.2009.06.012 19664444

[3] Dong XS Men Y Ringen K. Work-related injuries among Hispanic construction workers-evidence from the medical expenditure panel survey. Am J Indus Med. (2010) 53:561–9. 10.1002/ajim.20799 20187004

[4] Forst L Ahonen E Zanoni J Holloway-Beth A Oschner M Kimmel L. More than training: community-based participatory research to reduce injuries among hispanic construction workers. Am J Indus Med. (2013) 56:827–37. 10.1002/ajim.22187 23533016

[5] Economic News Release. Nonfatal Occupational Injuries and Illnesses Requiring Days Away From Work, 2007 (2008). Available online at: https://www.bls.gov/news.release/archives/osh2_11202008.pdf (accessed May 7, 2021).

[6] Centers for Disease Control. Work-Related Injury Deaths Among Hispanics–United States, 1992-2006. Centers for Disease Control and Prevention (2008). Available online at: http://www.cdc.gov/mmwr/preview/mmwrhtml/mm5722a1.htm (accessed January 1, 2015).

[7] Glazner JE Borgerding J Lowery JT Bondy J Mueller KL Kreiss K. Construction injury rates may exceed national estimates: evidence from the construction of Denver International Airport. Am J Indus Med. (1998) 34:105–12. 10.1002/(SICI)1097-0274(199808)34:2<105::AID-AJIM2>3.0.CO;2-W 9651619

[8] Leigh JP Du J McCurdy SA. An estimate of the U.S. government's undercount of nonfatal occupational injuries and illnesses in agriculture. Ann Epidemiol. (2014) 24:254–9. 10.1016/j.annepidem.2014.01.006 24507952 PMC6597012

[9] Valenzuela A Jr. Working on the margins: immigrant day labor characteristics and prospects for employment. Cent Comp Immigr Stud. (2000). Available online at: https://ccis.ucsd.edu/_files/wp22.pdf (accessed July 5, 2021).

[10] Gany F Novo P Dobslaw R Leng J. Urban occupational health in the Mexican and Latino/Latina immigrant population: a literature review. J Immigr Minority Health. (2014) 16:846–55. 10.1007/s10903-013-9806-8 23468371 PMC5469291

[11] Donato K Hakimzadeh S. The Changing Face of the Gulf Coast: Immigration to Louisiana, Mississippi, and Alabama. Migration Policy Institute. Available online at: https://www.migrationpolicy.org/article/changing-face-gulf-coastimmigration-louisiana-mississippi-and-alabama (accessed June, 2012).

[12] Fussell E. Post-Katrina New Orleans as a new migrant destination. Organ Environ. (2009) 22:458–69. 10.1177/1086026609347191

[13] Fussell E. Hurricane Chasers in New Orleans: Latino Immigrants as a Source of a Rapid Response Labor Force. Hisp J Behav Sci. (2009) 31:375–94. 10.1177/0739986309339735

[14] Brunette MJ. Construction safety research in the United States: targeting the Hispanic workforce. Injury Prevent. (2004) 10:244–8. 10.1136/ip.2004.005389 15314054 PMC1730115

[15] Brunette MJ. Development of educational and training materials on safety and health: targeting Hispanic workers in the construction industry. Fam Commun Health. (2005) 28:253–66. 10.1097/00003727-200507000-00006 15958883

[16] Schwatka NV Butler LM Rosecrance JR. An aging workforce and injury in the construction industry. Epidemiol Rev. (2011) 34:156–67. 10.1093/epirev/mxr020 22173940 PMC7735369

[17] Cuervo I Leopold L Baron S. Promoting community preparedness and resilience: a latino immigrant community–driven project following hurricane sandy. Am J Public Health. (2017) 107:S161–4. 10.2105/AJPH.2017.304053 28892443 PMC5594403

[18] Lewin S Dick J Pond P Zwarenstein M Aja GN van Wyk BE. Lay health workers in primary and community health care. Cochrane Database Syst Rev. (2005) 1:CD004015. 10.1002/14651858.CD004015.pub2 15674924

[19] Swider SM. Outcome effectiveness of community health workers: an integrative literature review. Public Health Nursing. (2002) 19:11–20. 10.1046/j.1525-1446.2002.19003.x 11841678

[20] Kines P Andersen LP Spangenberg S Mikkelsen KL Dyreborg J Zohar D. Improving construction site safety through leader-based verbal safety communication. J Safety Res. (2010) 41:399–406. 10.1016/j.jsr.2010.06.005 21059457

[21] Tuong W Larsen ER Armstrong AW. Videos to influence: a systematic review of ffectiveness of video-based education in modifying health behaviors. J Behav Med. (2014) 37:218–33. 10.1007/s10865-012-9480-7 23188480

[22] Wieland ML Nelson J Palmer T O'Hara C Weis JA Nigon JA. Evaluation of a tuberculosis education video among immigrants and refugees at an adult education center: a community-based participatory approach. J Health Commun. (2013) 18:343–53. 10.1080/10810730.2012.727952 23237382 PMC3577960

[23] Eubelen C Brendel F Belche J-L Freyens A Vanbelle S Giet D. Effect of an audiovisual message for tetanus booster vaccination broadcast in the waiting room. BMC Fam Pract. (2011) 12:104. 10.1186/1471-2296-12-104 21955570 PMC3196896

[24] Williams Q Jr Ochsner M Marshall E Kimmel L Martino C. The impact of a peer-led participatory health and safety training program for Latino day laborers in construction. J Saf Res. (2010) 41:253–61. 10.1016/j.jsr.2010.02.009 20630277

[25] de Souza RA Hecker S de Castro AB Stern H Hernandez A Seixas N. Novel approaches to development, delivery and evaluation of a peer-led occupational safety training for Latino day laborers. New Solut. (2012) 22:387–405. 10.2190/NS.22.3.i 22967369 PMC3654197

[26] Grzywacz JG Quandt SA Marín A Summers P Lang W Mills T. Occupational injury and work organization among immigrant Latino residential construction workers. Am J Indus Med. (2012) 55:698–706. 10.1002/ajim.22014 22266800

[27] Koskan AM Friedman DB Brandt HM Walsemann KM Messias DK. Preparing promotoras to deliver health programs for Hispanic communities: training processes and curricula. Health Promot Pract. (2013) 14:390–9. 10.1177/1524839912457176 22982761 PMC3876408

[28] Díaz Fuentes CM Martinez Pantoja L Tarver M Geschwind SA Lara M. Latino immigrant day laborer perceptions of occupational safety and health information preferences. Am J Indus Med. (2016) 59:476–85. 10.1002/ajim.22575 26901777

[29] Chávez V Israel B Allen AJ III DeCarlo MF Lichtenstein R Schulz A. A bridge between communities: video-making using principles of community-based participatory research. Health Promot Pract. (2004) 5:395–403. 10.1177/1524839903258067 15358912

[30] Lee SYD Stucky BD Lee JY Rozier RG Bender DE. Short assessment of health literacy—Spanish and English: a comparable test of health literacy for Spanish and English speakers. Health Serv Res. (2010) 45:1105–20. 10.1111/j.1475-6773.2010.01119.x 20500222 PMC2910571

[31] Napolitano M Lasarev M Beltran M Philips J Bryan C McCauley L. Un lugar seguro para sus ninos: development and evaluation of a pesticide education video. J Immigr Health. (2002) 4:35–45. 10.1023/A:1013059211540 16228753

[32] Kilanowski JF. Latino migrant farmworker student development of safety instructional videos for peer education. J Agromed. (2014) 19:150–61. 10.1080/1059924X.2014.894484 24911690

[33] Sokol R Fisher E. Peer support for the hardly reached: a systematic review. Am J Public Health. (2016) 106:e1–8. 10.2105/AJPH.2016.303180 27196645 PMC4984766

[34] Sanchez JI Briant KJ Wu-Georges S Gonzalez V Galvan A Cole S. Eat healthy, be active community workshops implemented with rural Hispanic women. BMC Womens Health. (2021) 21:24. 10.1186/s12905-020-01157-5 33435981 PMC7805196

[35] Swanberg JE Nichols HM Clouser JM Check P Edwards L Bush AM. A systematic review of community health workers' role in occupational safety and health research. J Immigr Minor Health. (2018) 20:1516–31. 10.1007/s10903-018-0711-z 29502238

[36] Caffaro F Micheletti Cremasco M Bagagiolo G Vigoroso L Cavallo E. Effectiveness of occupational safety and health training for migrant farmworkers: a scoping review. Public Health. (2018) 160:10–7. 10.1016/j.puhe.2018.03.018 29702273

[37] Menger LM Rosecrance J Stallones L Roman-Muniz IN. A guide to the design of occupational safety and health training for immigrant, latino/a dairy workers. Front Public Health. (2016) 4:282. 10.3389/fpubh.2016.00282 28066760 PMC5179979

